# Risk Factors of Relapse After Smoking Cessation: Results in China Family Panel Studies From 2010 to 2018

**DOI:** 10.3389/fpubh.2022.849647

**Published:** 2022-07-01

**Authors:** Naifan Hu, Zhenfan Yu, Yurun Du, Jiangping Li

**Affiliations:** ^1^Department of Epidemiology and Statistics, School of Public Health and Management, Ningxia Medical University, Yinchuan, China; ^2^Key Laboratory of Environmental Factors and Chronic Disease Control, Ningxia Medical University, Yinchuan, China

**Keywords:** relapse, smoking, quitting smoking, factors, competing risk, CFPS

## Abstract

**Background:**

Tobacco use is still highly prevalent globally in spite of the tobacco control efforts made by the governments. In view of the harm of smoking and relapse after smoking cessation, the purpose of this study is to establish a competitive risk model to determine potential risk factors for smoking relapse.

**Methods:**

The population-based cohort of ex-smokers over the age of 18 years was obtained from the China Family Panel Studies (CFPS) database from 2010 to 2018. Competing risk models were conducted to identify the risk factors for relapse.

**Results:**

A total of 1,019 subjects were included in this study, of which 311 (30.52%) subjects relapsed during the follow-up period. A multivariate analysis indicated that age < 40 years [hazard ratio (HR) 19.142; 95% CI: 10.641–34.434, *p* < 0.01], cohabitation (*HR*: 1.422; 95% *CI*: 1.081–1.87, *p* = 0.01), and often depression [*HR* 1.422; 95% *CI*, (1.081–1.87), *p* = 0.01] were associated with a great risk of relapse while the age of quitting smoking < 60 years (*HR*: 0. 436; 95% *CI*: 0.229–0.831, *p* < 0.01) and joining the Chinese Communist Party (CCP) (*HR* 0.611; 95% *CI*: 0.397–0.939, *p* = 0.03) were reduced risk factors for relapse.

**Conclusions:**

Approximately 3 in 10 ex-smokers were observed to relapse. There are various risk factors for relapse as well. In the face of such a serious situation, it is urgent to take action to control smoking.

## Introduction

Smoking remains a major public health concern, which is not only harmful to human health but also has a negative impact on society. It is estimated that 1 in 10 people have died from smoking globally (1). If no emergency interventions are taken, the number of deaths attributed to smoking is expected to rise to 8.3 million by 2030, with the largest increase in low-and middle-income countries, such as China and India (2). In a study of the birth cohort in the 1920's, smoking-related mortality among Asian men continued to rise, suggesting that smoking would remain a severe public health concern in most Asian countries over the next couple of decades (3). Smoking is connected with a reduction in sperm count and a rise in the number of sperm morphological defects as well (4). In addition, smoking has adverse effects on bones, joints, muscles, tendons, cartilage, ligaments (5), and so on. Smoking increases the risk of at least 17 human cancers and induces cell mutations and DNA methylation (6). What's worse, smoking is also associated with cardiovascular disease (CVD) (7), tuberculosis infection and death (8), gastroesophageal reflux disease (9), low quality of life, and depression (10).

The harm of smoking is so obvious that the World Health Organization (WHO) cooperates with countries around the world to formulate corresponding tobacco control measures. Under the joint efforts of various countries, the WHO launched the Framework Convention on Tobacco Control in February 2005, which aimed to control the tobacco epidemic and protect public health (11). In recent years, the pace of tobacco control in China has exceeded expectations, and there has been good progress in smoking cessation. Quitting smoking benefits all smokers, regardless of the age and amounts of smoking, which is an essential disease prevention strategy. Furthermore, there is indeed growing evidence that quitting smoking is effective in managing various diseases, such as reducing the risk of a variety of diseases, reducing disease mortality, and improving the course of disease (9, 12, 13).

Although the benefits of quitting smoking are apparent, there is no lack of relapse after quitting smoking. It is estimated that about 85% of ex-smokers will return to smoking within a year (14). Even after long-term quitting smoking, relapse is still possible (15). Considering that, we wonder whether those who have quit smoking will relapse and its factors, which will make a great difference in public health. Therefore, to achieve this goal, we intend to establish a model referring to competitive risk. Traditional survival analysis methods, such as standard Kaplan–Meier (KM) and Cox regression, cannot be employed in that they will overestimate the risk of relapse and produce competitive risk bias. So through the establishment of competitive risk model to explore the probability of relapse and other competitive events is feasible.

In this research, we aimed to comprehensively compare the characteristics of people who relapsed and who did not relapse, and we established a competitive risk model to determine the potential risk factors of smoking relapse in people who have given up smoking.

## Methods

### Data and Sampling

**Figure 1:**
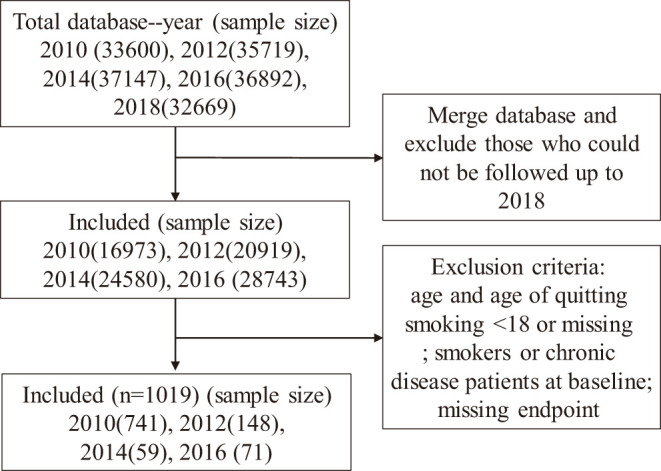
Sample screening flowchart. The study used a 2010-2018 CFPS database with exclusion criteria: 1. Participants who were in the cohort from 2010 to 2016 but could not be followed up to 2018; 2. Participants whose age and age of quitting smoking were less than 18 or missing; 3. Smokers and patients with chronic diseases in baseline years; 4. Participants with missing endpoint events. A total of 1019 participants were included.

The present research used the China Family Panel Studies (CFPS) database, a national and comprehensive database of social tracking projects that aims to reflect China's social, economic, demographic, educational, and health changes by tracking and collecting data at the individual, family, and community levels, providing a data basis for academic research and public policy analysis. Organized by the Institute of Social Sciences of Peking University since 2010, samples of CFPS have covered 25 provinces, accounting for 94.5% of the Chinese mainland's total population (16). To produce national and provincial representative samples, CFPS adopts the multi-stage stratified “probability proportional size” (PPS) sampling strategy, and implements a three-stage sampling process (www.isss.edu.cn/cfps/). All respondents read a statement explaining the purpose of the study and agreed to proceed. In this study, ex-smokers from 2010 to 2016 and followed up to 2018 were selected as subjects. A flow diagram of sample screening is shown in Figure 1.

### Methods and Model

Traditional survival analysis is generally concerned with only one outcome event and one duration (17), that is, regarding individuals who have died from other causes or lost follow-up as censored data, which will overestimate the cumulative incidence. As illustrated in this article, we explored whether ex-smokers would relapse first or develop chronic disease first, where the former was the result of our interest and the latter was regarded as competitive event. Therefore, the longitudinal data with competitive events similar to these should adopt the competitive risk model, which is a standardized structure of the multi-state model to deal with the survival data of a variety of end-point events and competitive risk events. It is very suitable for multiple outcomes, which can not only deal with the survival data of multiple potential results but also calculate the cumulative incidence function (CIF) of each result (18–20). The package CRRSTEP in R was applied to generate a CIF diagram, showing the estimated probability of smoking recrudesce over time.

The classical competitive risk model can be realized by a variety of models, such as the proportional hazard model, cause-specific hazard model, and sub-distribution hazard model, also known as Fine & Gray model as well (19). And what was utilized in this study is Fine and Gray model.

The sub-distribution hazard model also called as the cumulative risk model, uses the CIF to estimate the cumulative incidence of the target outcome event. CIF assumes that there is only one event type with expected attributes at a time, that is, the sum of the CIF of each category is equal to the compound event CIF.


$$\begin{array}{l} \text{Expression}:\text{CIF k }\left(\text{t}\right)\;=\text{ Pr }\left(\text{T}\leq \text{ t},\text{ D}=\text{k}\right) \end{array}$$

Where, CIF k (*t*) represents the probability of the k-th event before other events in time; and D represents the type of event that occurred. When there is a risk of competition, the outcome is no longer just survival and death, at this time *CIF*≠*F*() (21).

Proposed by Fine and Gray (22), the cumulative risk model can directly infer the effect of covariates on the cumulative incidence of type events. The model is based on


$$\begin{array}{l} \lambda_{k}^{CI}\;\left(t;Z\right)=\lambda_{k0}\left(t\right)\exp\left(Z^{T}\beta_{k}\right)\; \end{array}$$

(23),

Where $\lambda_{k}^{CI}\;\left(t;Z\right)=\frac{\underset{\Delta \to 0}{\lim}\mathrm{Pr}\left\{t≦T≦t+△,K=k|T\geq t\cup \left(T≦t\cap K\neq k\right);Z\right\}}{△}$. The sub-distribution hazard function $\lambda_{k}^{CI}\left(.\right)$ is the hazard function for the improper random variable T^*^ = *I*(*K* = *k*) × *T*+{1−*I*(*K* = *k*)} × ∞, where *I*(.) is an indicator function (24).

In contrast to Cox regression, the calculation of risk rate in this model takes into account not only the instantaneous incidence of the target outcome at the time but also the influence of the competitive risk outcome before the time.

### Relapse and Competing Events

Subjects who had answered the question “Age of quitting smoking” were initially defined as ex-smokers when enrolled at baseline. For this, two yes/no questions were separately used to evaluate whether one had relapsed or developed competing events, one was “Whether smoked cigarettes last month,” another was “Whether had doctor-diagnosed chronic disease in past 6 months.” For the first question, someone who answered “yes” was considered to have relapsed; if the second question's answer was “yes,” then the subject was considered to have developed a competitive event. On the basis of the sequence of event, we judged whether a person had relapsed or competed during follow-up. In other words, if relapse occurred first, then the outcome of the individual would be the end event, and the other events except this outcome were competitive events. If neither of the above conditions occurred, we defined it as a censored event. Survival time was defined as the difference between the onset of an end event or competitive event and the age at which people quit smoking.

### Covariates

Potential covariates included demographic characteristics: age (<40, 40–60, >60 years), gender, nationality (Han, others), education level (primary school or below, middle or technical secondary school, undergraduate or junior college), current marital status [unmarried, married, cohabitation (a couple living together without a marriage certificate or *de facto* marriage. Here refers to cohabitation without first marriage, also refers to cohabitation with first marriage), divorced/widowed], whether a member of the Chinese Communist Party (CCP), the number of children (0–2, >2), income status that was measured in the question “What is your income level in this area” (< ¥5,000, ≥¥5,000); psychological and behavioral characteristics: life satisfaction that was measured by the question “How would you rate your life satisfaction?” the question “How often during the past month did you feel depressed” was adopted to evaluate depression, fidget was measured by the question “How often during the past month did you feel restless or fidgety,” difficulty was measured through the question “How often during the past month did you feel that everything was an effort,” and meaninglessness that was measured by the question “How often during the past month did you feel that life was meaningless,” these indicators are divided by frequency (Almost none: Less than once a week, Sometimes: Once or two times a week, Often: Three or four times a week, Most of the time: More than five times a week); characteristics related to physical health: self-rated health status (excellent, good, fair, poor), quit smoking age (<40, 40–60, >60), and exercise was measured by “Frequency of your physical exercise last month when not on vacation,” whose options are divided into almost every day, sometimes (2 or 3 times a week and 2 or 3 times a month), almost none (once a month and never).

### Statistical Analysis

Statistical analysis and drawing were carried out with R4.0.5 software. The characteristics of relapse and non-relapse population were described by count and percentage, and were compared by the Pearson chi-square test for univariate analysis. In this sample, a considerable number of respondents developed chronic diseases before relapse. Nevertheless, the traditional Cox proportional hazard regression analysis does not study competitive events, which will lead to an overestimation of risk. Therefore, in the presence of competitive risk, Fine and Gray models were established to estimate the risk of relapse through R-packet CMPRSK, which was a multivariate analysis. CIF was used to show the probability of relapse over time. An interactive nomogram was established by R-Packet REGPLOT to visually analyze the CIF of individuals and identify high-risk groups.

## Results

### Characteristics of the Study Population

A total of 1,019 subjects were included in this study, of which 311 (30.52%) relapsed during follow-up period and competition events occurred in 43 (4.22%). We summarized the univariate analyzed results, finding that age, marital status, whether joining the CCP, education level, number of children, depression frequency, and exercise frequency were different in the relapsed and non-relapsed groups (*p* < 0.05). The distribution characteristics and univariate analysis of relapse and non-relapse population are shown in Table 1.

**Table 1:** Distribution characteristics and univariate analysis of relapse and non-relapse population.

**Variable/characteristic**	**Relapse *N* (%)**	**No-relapse *N* (%)**	**Overall *N* (%)**	***P*-value**
**Gender**				0.153
Male	292 (93.9%)	645 (91.1%)	937 (92.0%)	
Female	19 (6.1%)	63 (8.9%)	82 (8.0%)	
**Age**				<0.01
<40	117 (37.6%)	153 (21.6%)	270 (26.5%)	
40–60	155 (49.8%)	333 (47.0%)	488 (47.9%)	
>60	39 (12.5%)	222 (31.4%)	261 (25.6%)	
**Marital status**				<0.01
Unmarried	28 (9.0%)	35 (4.9%)	63 (6.2%)	
Cohabitation	3 (1.0%)	0 (0.0%)	3 (0.3%)	
In marriage	270 (86.8%)	628 (88.7%)	898 (88.1%)	
Divorced/Widowed	10 (3.2%)	45 (6.4%)	55 (5.4%)	
**Self-rated health status**				0.054
Excellent	31 (10.0%)	49 (6.9%)	80 (7.9%)	
Good	142 (45.7%)	298 (42.1%)	440 (43.2%)	
Fair	110 (35.4%)	292 (41.2%)	402 (39.5%)	
Poor	28 (9.0%)	69 (9.7%)	97 (9.5%)	
**Age of quitting smoking**				0.240
<40	162 (52.1%)	330 (46.6%)	492 (48.3%)	
40–60	131 (42.1%)	298 (42.1%)	429 (42.1%)	
>60	18 (5.8%)	80 (11.3%)	98 (9.6%)	
Nationality				0.789
Han	293 (94.2%)	670 (94.6%)	963 (94.5%)	
Others	18 (5.8%)	38 (5.4%)	56 (5.5%)	
**Education level**				0.016
Primary school or below	117 (37.6%)	323 (45.6%)	440 (43.2%)	
Middle or technical secondary school	170 (54.7%)	328 (46.3%)	498 (48.9%)	
Undergraduate or junior college	24 (7.7%)	57 (8.1%)	81 (7.9%)	
Whether joined CCP*				<0.01
No	283 (91.0%)	592 (83.6%)	875 (85.9%)	
Yes	28 (9.0%)	116 (16.4%)	144 (14.1%)	
**Number of children**				<0.01
0~2	247 (79.4%)	518 (73.2%)	765 (75.1%)	
>2	64 (20.6%)	190 (26.8%)	254 (24.9%)	
Income				0.116
<¥5,000	138 (44.4%)	336 (47.5%)	474 (46.5%)	
≥¥5,000	173 (55.6%)	372 (52.5%)	545 (53.5%)	
**Satisfaction with life**				0.114
Satisfaction	145 (46.6%)	379 (53.5%)	524 (51.4%)	
Fair	151 (48.6%)	302 (42.7%)	453 (44.5%)	
Dissatisfaction	15 (4.8%)	27 (3.8%)	42 (4.1%)	
**Depression frequency**				<0.01
Most of the time	6 (1.9%)	8 (1.1%)	14 (1.4%)	
Often	21 (6.8%)	55 (7.8%)	76 (7.5%)	
Sometimes	138 (44.4%)	235 (33.2%)	373 (36.6%)	
Almost none	146 (46.9%)	410 (57.9%)	556 (54.6%)	
**Fidget frequency**				0.543
Most of the time	5 (1.6%)	10 (1.4%)	15 (1.5%)	
Often	23 (7.4%)	42 (5.9%)	65 (6.4%)	
Sometimes	88 (28.3%)	193 (27.3%)	281 (27.6%)	
Almost none	195 (62.7%)	463 (65.4%)	658 (64.6%)	
**Difficulty frequency**				0.063
Most of the time	7 (2.3%)	15 (2.1%)	22 (2.2%)	
Often	22 (7.1%)	49 (6.9%)	71 (7.0%)	
Sometimes	114 (36.7%)	211 (29.8%)	325 (31.9%)	
Almost none	168 (54.0%)	433 (61.2%)	601 (59.0%)	
**Meaningless frequency**				0.318
Most of the time	6 (1.9%)	8 (1.1%)	14 (1.4%)	
Often	12 (3.9%)	19 (2.7%)	31 (3.0%)	
Sometimes	35 (11.3%)	96 (13.6%)	131 (12.9%)	
Almost none	258 (83.0%)	585 (86.2%)	843 (82.7%)	
**Exercise frequency**				0.035
Almost every day	10 (3.2%)	33 (4.7%)	43 (4.2%)	
Sometimes	88 (28.3%)	246 (34.7%)	334 (32.8%)	
Almost none	213 (68.5%)	429 (60.6%)	642 (63.0%)	

^*^CCP, Chinese Communist Party.

**Figure 2:**
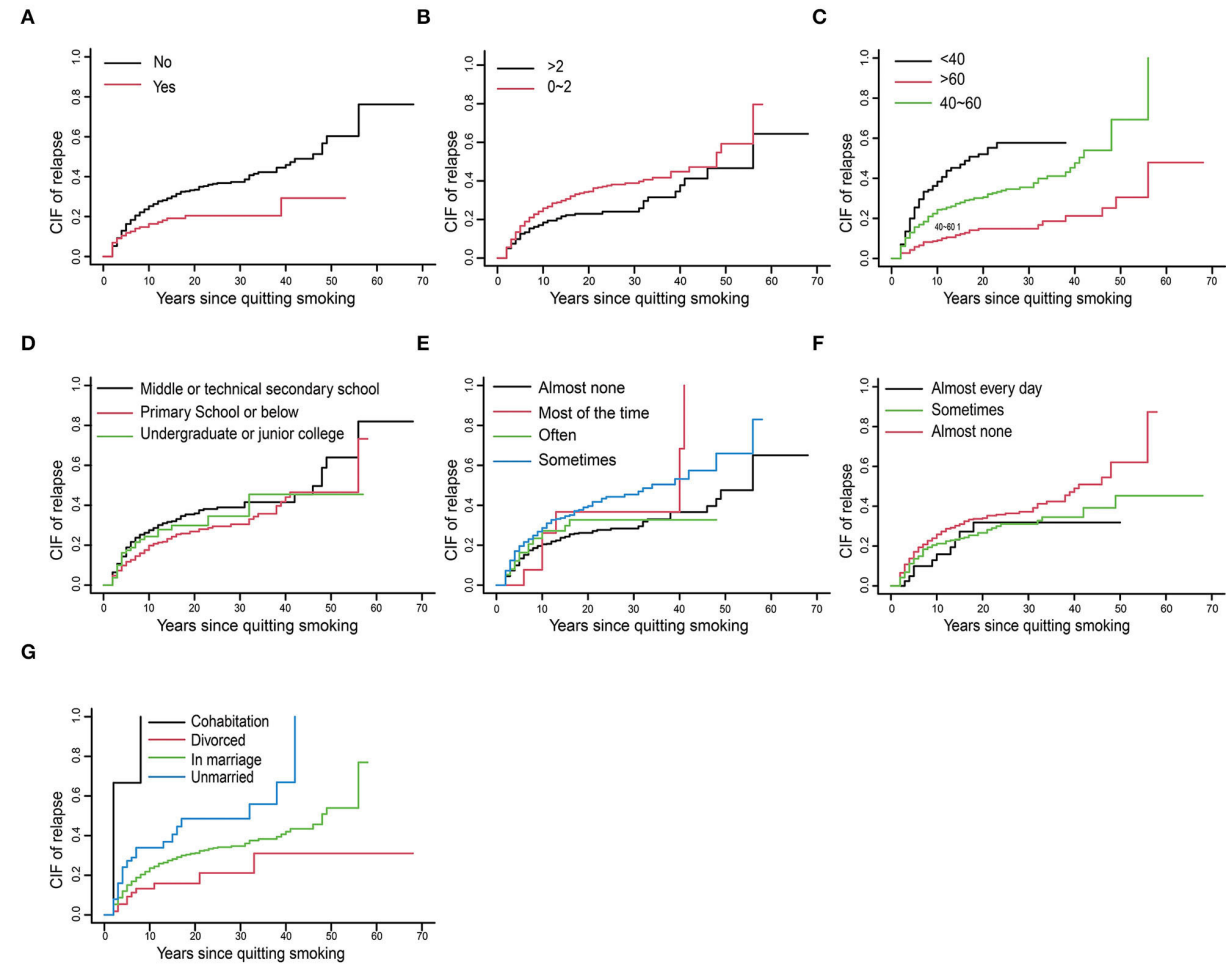
The cumulative incidence function (CIF) of relapse in smokers with relapse as the end event. **(A)** Whether joined Chinese communist party. **(B)** Number of children. **(C)** Age. **(D)** Education level. **(E)** Depression frequency. **(F)** Exercise frequency. **(G)** Marital status.

Based on the results of univariate analysis, we drew the CIF curve of relapse to express the research results more intuitively in Figure 2 *(The cumulative incidence function (CIF) of relapse in smokers with relapse as the end event)*. It is shown in Figure 2A that not joining CCP had a higher cumulative incidence of smoking relapse than those who had joined CCP. As shown in Figure 2B, the more children there were, the lower the cumulative incidence of smoking relapsed. It was depicted in Figure 2C that the younger the age there was, the higher the cumulative incidence of smoking relapse would be. As shown in Figure 2D, people with a high cumulative incidence of smoking relapse tended to have a lower education level. As illustrated in Figure 2E, people who barely felt depressed had a lower cumulative incidence of relapse. As graphically displayed in Figure 2F, people exercising almost none had a higher cumulative relapse rate. The difference in marital status was presented in Figure 2G. People who were unmarried or in cohabitation were more likely to relapse, higher than in marriage and divorced.

### Risk Factors for Developing Relapse

Further analysis of multivariate competitive risk showed that five variables were retained in the final optimization model, such as age, quitting smoking age, CCP, marital status, and depression as shown in Table 2. The hazard ratio (*HR*) for under the age of 40 against more than 60 was 19.142 (95% *CI*: 10.641–34.434, *p* < 0.01), that was the younger people were, the higher their risk of relapse. The age of quitting smoking <60 years was a reduced risk factor for relapse (*HR*: 0. 436; 95% *CI*: 0.229–0.831, *p* < 0.01). Joining the CCP was a protective risk of relapse (*HR* 0.611; 95% *CI*: 0.397–0.939, *p* = 0.03). Cohabitation (*HR*: 9.982; 95% *CI*: 4.077–24.441, *p* < 0.01) was identified to be in connection with a higher risk of relapse. With respect to depression frequency, depression often was a risk factor for relapse (*HR*: 1.422; 95% *CI*: 1.081–1.87, *p* = 0.01).

**Table 2:** Multivariable competing risk analysis for relapse.

**Variable**	**HR**	**95% CI**	***p*-value**
**Age**			
<40	19.142	(10.641, 34.434)	<0.01
40–60	4.504	(2.821, 7.193)	<0.01
>60	Reference		
**Age of quitting smoking**			
<40	0.104	(0.051, 0.214)	<0.01
40–60	0.436	(0.229, 0.831)	0.01
>60	Reference		
Whether joined CCP*			
Yes	0.611	(0.397, 0.939)	0.03
No	Reference		
**Gender**			
Male	1.473	(0.922, 2.353)	0.10
Female	Reference		
**Nationality**			
Han	0.971	(0.595, 1.587)	0.91
Others	Reference		
**Education level**			
Undergraduate or junior college	0.870	(0.522, 1.448)	0.59
Middle or technical secondary school	1.231	(0.944, 1.605)	0.13
Primary school or below	Reference		
**Self-rated health status**			
Excellent	1.362	(0.753, 2.463)	0.31
Good	0.991	(0.602, 1.631)	0.97
Fair	0.827	(0.506, 1.352)	0.45
Poor	Reference		
**Marital status**			
Unmarried	1.596	(0.78, 3.267)	0.20
In marriage	1.449	(0.784, 2.679)	0.24
Cohabitation	9.982	(4.077, 24.441)	<0.01
Divorced/Widowed	Reference		
**Number of children**			
0–2	0.994	(0.728, 1.358)	0.97
>2	Reference		
**Income**			
<¥5,000	1.150	(0.904, 1.465)	0.26
≥¥5,000	Reference		
**Satisfaction with life**			
Satisfaction	0.936	(0.534, 1.639)	0.82
Fair	0.974	(0.564, 1.682)	0.93
Dissatisfaction	Reference		
**Depression frequency**			
Most of the time	1.320	(0.574, 3.035)	0.51
Often	1.422	(1.081, 1.87)	0.01
Sometimes	0.905	(0.541, 1.514)	0.70
Almost none	Reference		
**Fidget frequency**			
Most of the time	0.765	(0.3, 1.952)	0.58
Often	0.875	(0.655, 1.169)	0.37
Sometimes	1.064	(0.662, 1.71)	0.80
Almost none	Reference		
**Difficulty frequency**			
Most of the time	1.094	(0.45, 2.662)	0.84
Often	1.270	(0.954, 1.689)	0.10
Sometimes	0.898	(0.507, 1.591)	0.71
Almost none	Reference		
**Meaningless frequency**			
Most of the time	1.958	(0.846, 4.534)	0.12
Often	0.858	(0.581, 1.267)	0.44
Sometimes	1.809	(0.825, 3.965)	0.14
Almost none	Reference		
**Exercise frequency**			
Almost every day	0.575	(0.282, 1.173)	0.13
Sometimes	0.876	(0.669, 1.147)	0.34
Almost none	Reference		

^*^CCP, Chinese Communist Party.

### Interactive Nomogram for Predictive Cumulative Incidence

**Figure 3:**
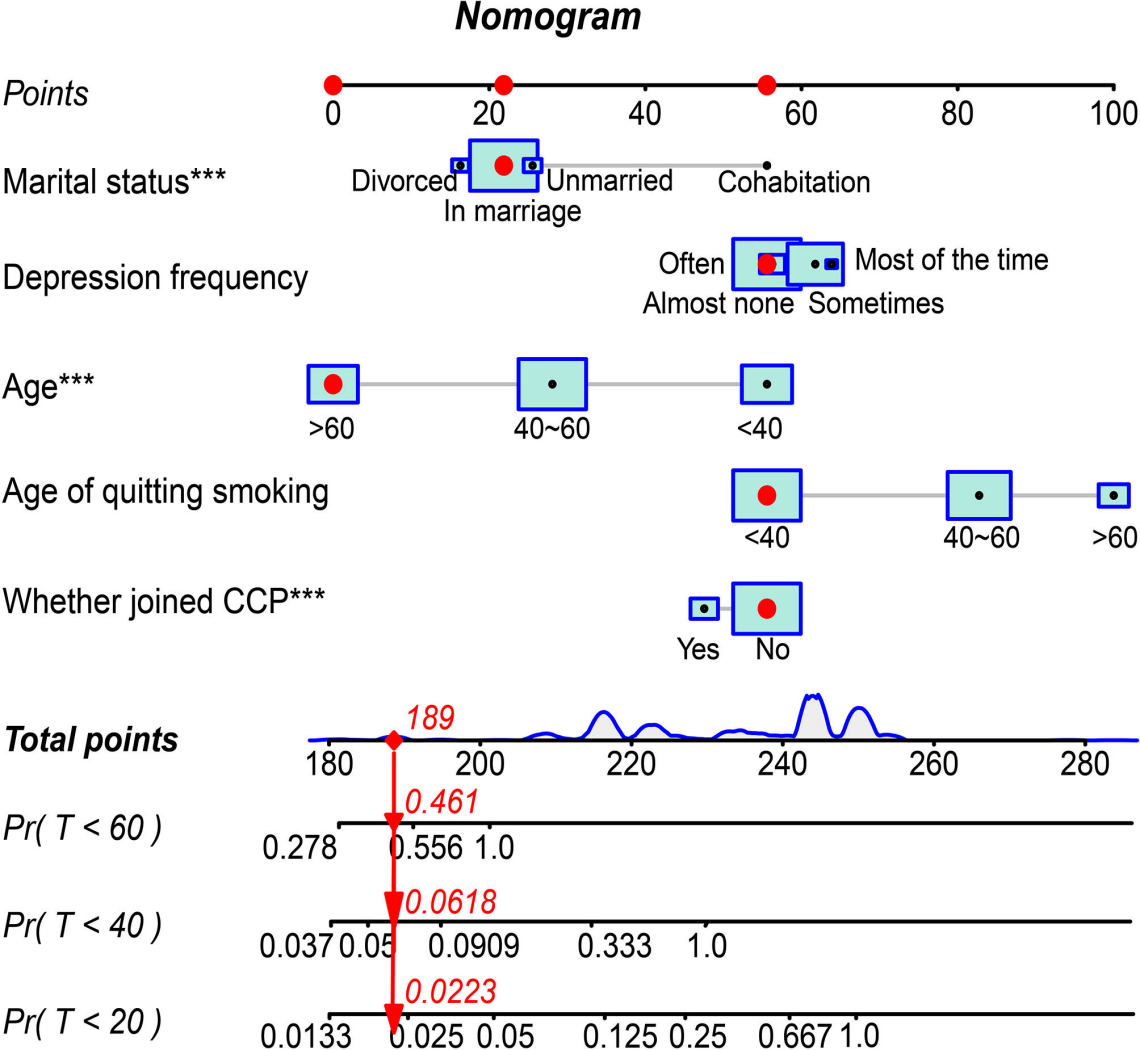
Interactive Nomogram visualizing the cumulative incidence of relapse at 20th, 40th, and 60th years in the competitive risk model.

Based on the predictors above, an interactive nomogram was generated to estimate the 20-, 40-, and 60-year cumulative incidences of relapse personally after smoking cessation. The scoring with the nomogram in Figure 3 effectively discriminated the risk of relapse: age, marital status, and CCP emerged as the strongest predictors. In the nomogram, the values of each covariate of patients with ID = 189 in the dataset were mapped to the corresponding scores, and the total scores were calculated, and the cumulative recurrence probabilities at 20, 40, and 60th years were calculated, respectively. This probability is the cumulative recurrence probability after controlling the competition risk, which is 0.0223, 0.0618, and 0.461, respectively.

## Discussion

After screening four waves of 1,019 subjects from CFPS database and following up to 2018, 30.52% relapsed. Considering that ~3 in 10 ex-smokers were observed to relapse, intensive surveillance in all ex-smokers seemed to be impractical. Therefore, a prediction tool to identify ex-smokers who may relapse is an urgent need. In our present study, an interactive nomogram based on competing risk model was proposed as a convenient tool to determine the high-risk ex-smokers. Under the age of 60 years, cohabitation and depression were associated with a great risk of relapse, while the age of quitting smoking <60 years and belonging to the CCP were deemed to be reduced risk factors for relapse.

As for the influencing factors of age, the results showed that the younger the age, the greater the risk of smoking relapse would be. It is well established that people under the 60 years of age are at a critical stage in their lives, when they need to take care of family and career at the same time. However, life and work stress (25) occur when balancing the relationship, it is possible for people to relieve stress through smoking. Maybe the young are more addicted to smoking as a consequence of depression (7, 26, 27) and stress (25, 28, 29) from family, society, or emotion dysregulation (30, 31). Implementing the decompression of the whole society, resisting the temptation of tobacco and developing good living habits can help reduce relapse to a certain extent.

The influence of quitting smoking age is contrast with the effect of age on relapse smoking. The risk ratio between the age of quitting smoking between 40 and 60 years old and the age of quitting smoking over 60 years old was 0.436. That was the older the quitting smoking age was, the greater the risk of relapse would be. People who quit smoking after the age of 60 had longer years of smoking and there was a study suggesting that the longer the smoking age is, the more addicted people would be (32), making it more difficult to quit and easier to relapse.

A Japanese study (33) found that cohabiters smoked more, which was consistent with our findings. Cohabitation is a major influencing factor of relapse, although adults face the pressure of life at this time, unlike being married, they only need to deal with the relationship and work. Couples are happy just to live together, but over a long period of time, the shortcomings of both sides are all exposed, and the pressure caused by emotional or work problems will cause people to relapse.

Little evidence manifests that there is a direct relationship between whether joining the CCP and tobacco. Compared with others, joining the CCP had an inverse association with relapse. Some studies in China have shown that members of the CCP have a higher awareness of social morality than those who do not belong to the CCP (34), and the central government has issued relevant regulations to take the lead in banning smoking in public places for civil servants (35), which has aroused social concern. Therefore, compared with other people, party members are less likely to recur smoking, in addition to the measures imposed on them by the state, they have stronger willpower and beliefs. In addition, people those who had joined the CCP had higher cognitive abilities than others, thus, had a higher awareness of health and the hazards of smoking.

There is no doubt that depression is a factor affecting relapse. Most studies show a strong link between depression and smoking as well (36, 37). According to the frequency of depression, we divided it into four stages. The results showed that those who choose “often” were the most likely to relapse. Maybe in the first two stages, people had a low degree of depression and can vent their emotions in other ways. Meanwhile, studies have found that smoking can be used as a way to relieve stress and a protective factor for depression (38, 39). However, those who choose “most of the time” might have more severe depression and receive treatment. So, the results of this study showed that people who often felt depressed had a 0.422 times higher risk than those who rarely felt depressed.

Smoking and smoking cessation are both slow processes. Especially when smoking for a long time, nicotine and other harmful substances (40) gradually infringe upon the body of smokers, which not only makes smokers dependent on it, but also causes grievous damage (41–43). Hence, it is difficult for us to imagine the secondary harm caused by relapse. Tobacco control action brooks no delay, which is not only the responsibility of the whole society, but also closely related to every one of us. Starting with the risk factors of smoking to reduce the high-risk factors and remove the low risk factors is one of the strategies for tobacco control action. Risk factors mentioned in this study, such as age, age of quitting smoking, and cohabitation can provide suggestions for the implementation of tobacco control programs.

Although the CFPS database is nationally representative in China, it is inevitable that there is bias in the survey process, and there is always a sampling error in the sample representation of the population. Second, the questionnaires in different years are constantly changing, so that some variables do not exist in some years. In addition, the respondents' answers to the questionnaire were subjective and there was no specific clinical diagnosis or proof, thus, there is a deviation in the accuracy of questions and answers. Besides, since we performed a secondary analysis of the data, other confounding factors that might influence relapse were not included. Since our study was still observational, causal inference was not strong enough. When it came to defining the time of smoking and quitting, we could only track their years, and cannot determine the specific date, which may have some error.

## Conclusion

Approximately 3 in 10 ex-smokers was observed to relapse. Risk factors for relapse are varied as well. In the face of such a serious situation, it is urgent to take action to control smoking.

## Data Availability Statement

Publicly available datasets were analyzed in this study. This data can be found at: https://opendata.pku.edu.cn.

## Ethics Statement

Written informed consent was obtained from the individual(s) for the publication of any potentially identifiable images or data included in this article.

## Author Contributions

NH conducted the literature search, did the data extraction and quality assessment, which was checked by JL. YD contributed to the analysis and write-up of findings. NH wrote the first draft of the manuscript. ZY and JL contributed to further drafts. All authors were involved in the design of the study. All authors contributed to the article and approved the submitted version.

## Funding

This work was supported by the research project of Ningxia Medical University (No. XT2019009).

## Conflict of Interest

The authors declare that the research was conducted in the absence of any commercial or financial relationships that could be construed as a potential conflict of interest.

## Publisher's Note

All claims expressed in this article are solely those of the authors and do not necessarily represent those of their affiliated organizations, or those of the publisher, the editors and the reviewers. Any product that may be evaluated in this article, or claim that may be made by its manufacturer, is not guaranteed or endorsed by the publisher.
